# A strong constitutive ethylene-response phenotype conferred on Arabidopsis plants containing null mutations in the ethylene receptors *ETR1 *and *ERS1*

**DOI:** 10.1186/1471-2229-7-3

**Published:** 2007-01-15

**Authors:** Xiang Qu, Brenda P Hall, Zhiyong Gao, G Eric Schaller

**Affiliations:** 1 Department of Biochemistry, University of New Hampshire, Durham, NH 03824, USA; 2 Department of Biological Sciences, Dartmouth College, Hanover, NH 03755, USA; 3 Current affiliation : California Institute of Technology, Biology Dept., Pasadena, CA 91125, USA

## Abstract

**Background:**

The ethylene receptor family of Arabidopsis consists of five members, falling into two subfamilies. Subfamily 1 is composed of ETR1 and ERS1, and subfamily 2 is composed of ETR2, ERS2, and EIN4. Although mutations have been isolated in the genes encoding all five family members, the only previous insertion allele of *ERS1 *(*ers1-2*) is a partial loss-of-function mutation based on our analysis. The purpose of this study was to determine the extent of signaling mediated by subfamily-1 ethylene receptors through isolation and characterization of null mutations.

**Results:**

We isolated new T-DNA insertion alleles of subfamily 1 members *ERS1 *and *ETR1 *(*ers1-3 *and *etr1-9*, respectively), both of which are null mutations based on molecular, biochemical, and genetic analyses. Single mutants show an ethylene response similar to wild type, although both mutants are slightly hypersensitive to ethylene. Double mutants of *ers1-3 *with *etr1-9*, as well as with the previously isolated *etr1-7*, display a constitutive ethylene-response phenotype more pronounced than that observed with any previously characterized combination of ethylene receptor mutations. Dark-grown *etr1-9;ers1-3 *and *etr1-7;ers1-3 *seedlings display a constitutive triple-response phenotype. Light-grown *etr1-9;ers1-3 *and *etr1-7;ers1-3 *plants are dwarfed, largely sterile, exhibit premature leaf senescence, and develop novel filamentous structures at the base of the flower. A reduced level of ethylene response was still uncovered in the double mutants, indicating that subfamily 2 receptors can independently contribute to signaling, with evidence suggesting that this is due to their interaction with the Raf-like kinase CTR1.

**Conclusion:**

Our results are consistent with the ethylene receptors acting as redundant negative regulators of ethylene signaling, but with subfamily 1 receptors playing the predominant role. Loss of a single member of subfamily 1 is largely compensated for by the activity of the other member, but loss of both subfamily members results in a strong constitutive ethylene-response phenotype. The role of subfamily 1 members is greater than previously suspected and analysis of the double mutant null for both *ETR1 *and *ERS1 *uncovers novel roles for the receptors not previously characterized.

## Background

The simple gas ethylene serves as a diffusible hormone in plants [1,2]. Ethylene regulates seed germination, seedling growth, leaf and petal abscission, organ senescence, fruit ripening, and responses to stress and pathogens. Mutants affecting ethylene responses have been isolated in Arabidopsis, and characterization of these mutants has led to the identification of ethylene receptors and several downstream components in the ethylene signal transduction pathway [3-5].

The Arabidopsis ethylene receptor family consists of five members: ETR1, ERS1, ETR2, ERS2 and EIN4 [6,4,7]. The ethylene receptors have similar overall structures with transmembrane domains near their N-termini and putative signaling motifs in their C-terminal halves, but can be divided into two subfamilies based on phylogenetic analysis and some shared structural features [3,6,4]. All five receptor members contain three highly conserved transmembrane domains that incorporate the ethylene binding site, and a GAF domain of unknown function in their N-terminal halves [7-10]. The subfamily 1 receptors ETR1 and ERS1 have a highly conserved histidine kinase domain containing all the required motifs essential for kinase functionality, with histidine kinase activity for both having been demonstrated in vitro [11,12]. The subfamily 2 receptors ETR2, ERS2, and EIN4 lack residues considered essential for histidine kinase activity and have instead been proposed to act as serine/threonine kinases [12]. Some of the ethylene receptors (ETR1, ETR2 and EIN4) possess a receiver domain in addition to a histidine kinase-like domain.

To define role of the receptors in signaling, loss-of-function mutations were initially isolated in four out of the five Arabidopsis ethylene receptors, no mutation being isolated for ERS1 [13]. Loss-of-function (LOF) mutations in any single ethylene receptor demonstrated little or no effect upon seedling growth, consistent with functional overlap within the receptor family. Plants with multiple LOF mutations in the receptors demonstrated a constitutive ethylene response, indicating that the receptors are negative regulators of ethylene signaling [13]. These effects of receptors upon signaling are apparently due to their physical association with the Raf-like kinase CTR1 [14-16]. According to the current model, CTR1 actively suppresses ethylene responses in the air (absence of ethylene). Ethylene binding by the receptors results in a conformational change in CTR1, reducing its kinase activity and relieving repression of the ethylene response pathway. Because CTR1 is physically associated with the receptors, loss of a sufficient number of ethylene receptors, such as occurs with the higher order LOF mutations, results in a redistribution of CTR1 from the membrane to the soluble fraction [15]. Without membrane localization, CTR1 is apparently unable to suppress the ethylene responses, which results in a constitutive ethylene response phenotype.

Recently, a T-DNA insertion allele into the 5' untranslated region of *ERS1 *was identified and this mutant allele named *ers1-2 *[17-19]. The responsiveness to ethylene of the *ers1-2 *mutant plants was similar to that of wild-type plants. But when combined with an *etr1-7 *LOF mutation, the *etr1-7*;*ers1-2 *double mutant displayed an ethylene response phenotype when grown in the absence of ethylene, this effect being more pronounced in light-grown plants than in dark-grown seedlings. Although these data reveal a significant role for the subfamily 1 receptors in signaling, the degree to which they contribute is unclear because transcript for *ers1-2 *could be detected[17,18]. Here we report on the isolation and analysis of two new T-DNA insertion mutant alleles of *ETR1 *and *ERS1*, named *etr1-9 *and *ers1-3*. Our results indicate that both *etr1-9 *and *ers1-3 *are null alleles for their genes, whereas *ers1-2 *is a partial LOF allele. Analysis of the *etr1-7;ers1-3 *and *etr1-9;ers1-3 *double mutants indicates that the subfamily 1 members ETR1 and ERS1 play more predominant roles in the regulation of ethylene responses in Arabidopsis than previously suspected.

## Results

### Isolation of the *ers1-3 *and *etr1-9 *T-DNA insertion mutations

**Figure 1:**
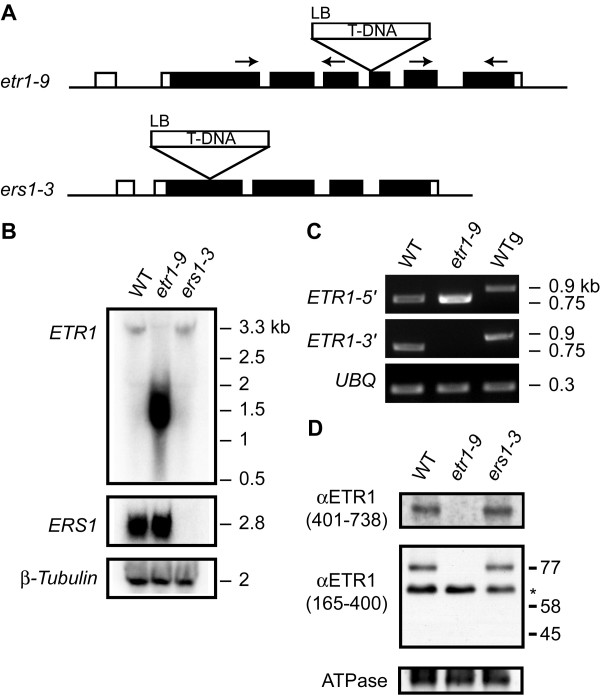
Analysis of the *ers1-3 *and *etr1-9 *T-DNA insertion alleles. (A) Positions of T-DNA insertions. Arrows indicate site of primers used for RT-PCR analysis. (B) Northern blot analysis. Poly-A RNA from wild type (WT), *etr1-9*, and *ers1-3 *were probed for the expression of *ETR1*, *ERS1*, or the control β-tubulin. (C) RT-PCR analysis for expression from *ETR1*. Expression was analyzed in wild type (WT) and in *etr1-9 *backgrounds using primers specific for sequences 5' and 3' to the site of the T-DNA insertion. Genomic DNA (WTg) was included to confirm the difference in PCR product sizes from cDNA and genomic DNA templates. Ubiquitin (*UBQ*) was used as a control. (D) Immunoblot analysis for expression of ETR1. Membranes from wild type and mutant lines were probed with antibodies against different regions of ETR1, anti-ETR1(165–401) and anti-ETR1(401–738), as well as with the anti-(H^+^-ATPase) antibody as a loading control. Wild-type ETR1 migrates at a molecular mass of 77 kDa. The asterisk indicates a nonspecific protein of 65 kDa that cross reacts with the anti-ETR1(165–401) antibody but not with anti-ETR1(401–738) antibody. The predicted migration position of the hypothetical truncated receptor (58 kDa) is indicated.

To obtain additional T-DNA insertion mutations in *ERS1 *and *ETR1*, we screened the Wisconsin Basta population representing 72,960 T-DNA insertion lines from the Arabidopsis Knockout Facility [20]. A line was identified that contained a T-DNA insertion within the first exon of *ERS1 *(Fig. 1A). Sequence at the T-DNA junction with *ERS1 *was ATACTATTTTAAGAACCACaatgagtaaata(taaatggcgacatgtccggg), with capitals indicating *ERS1 *sequence and parentheses indicating T-DNA left border sequence. This mutation was named *ers1-3 *to differentiate it from the previously isolated *ers1-2 *insertion mutation [17,18]. Northern blot analysis indicated an absence of *ERS1 *transcript in the *ers1-3 *background, consistent with *ers1-3 *being a complete null allele (Fig. 1B). Western-blot analysis demonstrated that the expression level of ETR1 remained the same in both the wild-type and *ers1-3 *backgrounds, suggesting that ETR1 did not functionally compensate for the loss of ERS1 (Fig. 1D), functional compensation having been found in some cases upon elimination of ethylene receptors in tomato [21].

From the same T-DNA population, we also identified a T-DNA insertion allele of *ETR1*, which we named *etr1-9 *to differentiate it from the previously identified *etr1-5*, *etr1-6*, *etr1-7*, and *etr1-8 *LOF mutants [13]. The T-DNA was inserted into the fourth exon of ETR1 (Fig. 1A). Sequence at the T-DNA junction with *ETR1 *was GGTAAAAGACTCTGGAGCtcca, with capitals indicating *ETR1 *sequence and lower case indicating random sequence between *ETR1 *and left border of T-DNA. Northern blot analysis indicated that no full-length *ETR1 *message was made in the *etr1-9 *mutant seedlings (Fig. 1B). However, a highly expressed truncated transcript was found at a lower molecular weight. RT-PCR analysis demonstrated that this transcript originated from the 5' end of the gene, prior to the site of the T-DNA insertion (Fig. 1C). To determine whether any truncated protein was made, immunoblot analysis was performed and no full-length or truncated ETR1 receptor was detected (the hypothetical truncated ETR1 protein being predicted to consist of 515 amino acids with a molecular weight of 58 kD) (Fig. 1D). Therefore, *etr1-9 *is a null mutation of *ETR1*. The increased expression of the truncated transcript from the native promoter may arise as part of compensatory mechanism due to lack of signaling by ETR1. The *etr1-9 *allele is in the WS background, the same as the *ers1-2 *and *ers1-3 *mutant alleles, and is thus more suitable for genetic crosses than the previous LOF mutant alleles of *ETR1*, which are in the Columbia background.

### Quantitative analysis of the ethylene-induced seedling growth response in single *etr1 *and *ers1 *T-DNA insertion mutants

**Figure 2:**
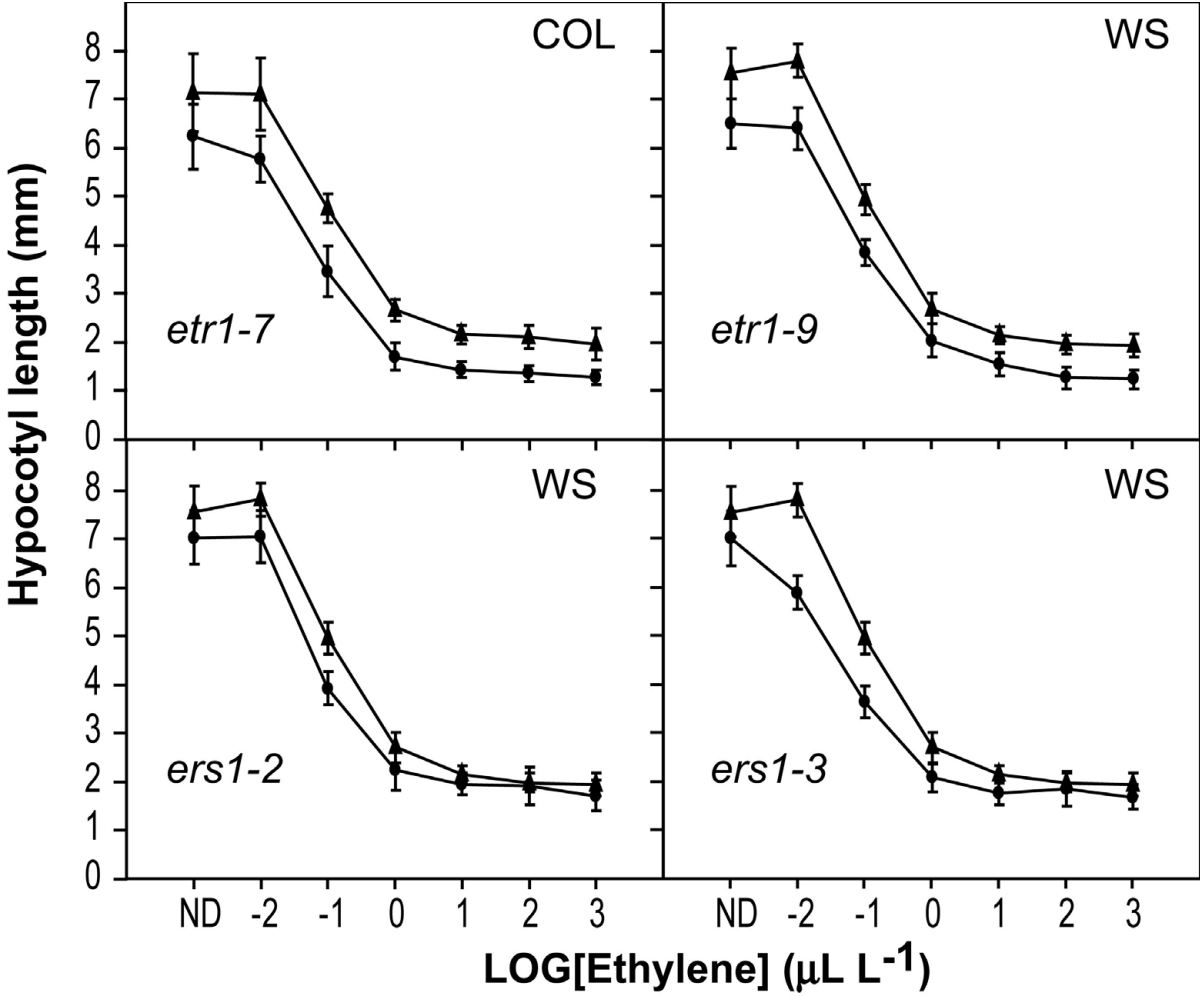
Ethylene dose-response analysis for *etr1 *and *ers1 *single mutants. The *etr1-7 *mutant is of ecotype Columbia (COL). The *etr1-9*, *ers1-2*, and *ers1-3 *mutants are of the ecotype Wassilewskija (WS). The response of the hypocotyl length to ethylene is shown for the mutants (black circles), with the appropriate wild-type control included for comparison (black triangles). Values represent the means with standard deviation for at least 25 measurements. ND, No detectable ethylene. AVG (5 μM) was included in growth media to inhibit ethylene biosynthesis by the seedlings.

Both *etr1-9 *and *ers1-3 *T-DNA insertion mutants displayed a wild-type-like growth phenotype. To gain quantitative information, ethylene dose response analyses were performed for the T-DNA insertion mutants *ers1-2*, *ers1-3*, and *etr1-9 *in the WS background and for the *etr1-7 *LOF mutant in the Columbia background (Fig. 2). Homozygous etiolated seedlings were grown in air and in ethylene at different concentrations ranging from 0 to 1000 μL L^-1^. The inhibitor aminoethylvinyl-glycine (AVG) was included in growth media to inhibit ethylene biosynthesis by seedlings. The *etr1-9 *mutant seedlings displayed reduced hypocotyl elongation in comparison with the wild-type WS control at all tested ethylene concentrations, which is consistent with dose-response analysis of the *etr1-7 *loss-of-function mutant (Fig. 2; [13]). This effect of the *etr1-7 *mutation upon growth has been demonstrated to be due to enhanced ethylene responsiveness of the seedling [22]. We confirmed the enhanced responsiveness of *etr1-9 *by treating the seedlings with 100 μM AgNO_3_, which inhibits ethylene perception by the receptors. The hypocotyl length of 4-day-old etiolated seedlings of *etr1-9 *was similar to wild type with Ag-treatment [10.25 mm (SD = 1.24) for *etr1-9 *compared to 10.54 mm (SD = 1.18) for wild type) but was significantly shorter than wild type when grown on AVG [9.25 mm (SD = 1.03) for *etr1-9 *compared to 10.25 mm (SD = 1.78)]. The *etr1-9 *mutant exhibited a 10% reduction in hypocotyl length comparing the AVG to Ag treatments, whereas wild type exhibited only a 3% reduction in hypocotyl length. As found with *etr1-7*, this demonstrates that *etr1-9 *requires ethylene perception for manifestation of the shortened hypocotyl phenotype [22]. The shortened hypocotyl of *etr1-9 *found with AVG treatment would be due to the low levels of endogenous ethylene production not eliminated by the treatment with this ethylene biosynthesis inhibitor.

The *ers1-3 *mutant was similar to wild type in the absence of exogenous ethylene (Fig. 2). But *ers1-3 *exhibited a growth response to 0.01 μL/L ethylene, indicating a greater sensitivity to ethylene than wild type which did not show a response at this ethylene concentration. In addition, the ethylene-responsiveness of the *ers1-3 *mutant was slightly greater than that of *ers1-2*, this difference potentially arising due to the presence of low levels of the *ERS1 *transcript in the *ers1-2 *background but lacking in the *ers1-3 *background [17,18]. The difference in the ethylene responses between *ers1-3 *and *etr1-9*, particularly when examined without exogenous ethylene treatment, is likely due to difference in their expression, *ETR1 *being constitutively expressed but *ERS1 *being induced by ethylene. These data indicate that single LOF mutations in either *ETR1 *or *ERS1 *result in some hypersensitivity to ethylene.

### Dark-grown seedlings of the *etr1-7;ers1-3 *and *etr1-9;ers1-3 *double mutants display a strong constitutive triple-response phenotype

**Figure 3:**
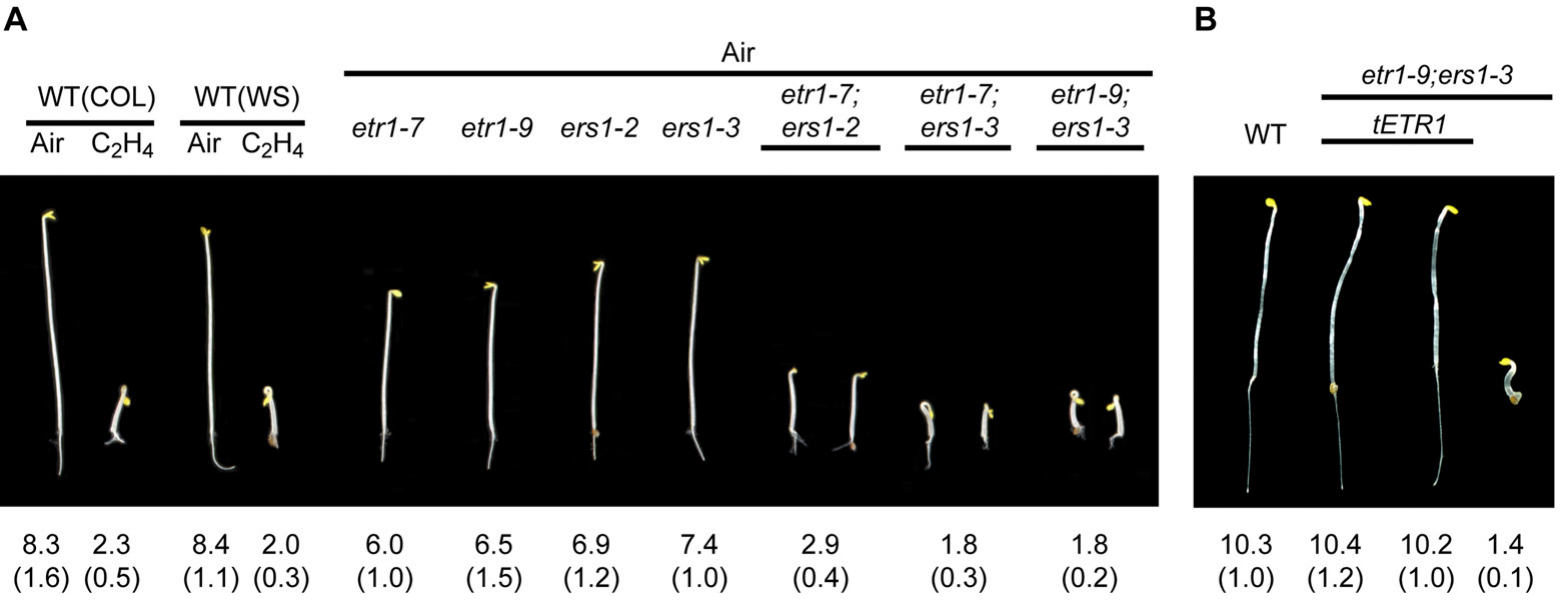
The *etr1-7;ers1-3 *and *etr1-9;ers1-3 *mutants exhibit a severe constitutive ethylene-response phenotype in dark-grown seedlings. (A)Effect of single and double mutations of subfamily 1 receptors upon seedling growth. *etr1-7 *was isolated from ecotype Columbia (Col), whereas *etr1-9*, *ers1-2*, and *ers1-3 *were isolated from ecotype Wassilewskija (WS). Both Col and WS wild-type seedlings are included as controls. Seedlings were grown in the dark for 3.5 days in the absence (air) or in the presence of ethylene (10 μL/L). AVG (5 μM) was included in growth media to inhibit ethylene biosynthesis by the seedlings. Mean hypocotyl lengths are given in millimeters with SD in parentheses. (B) Rescue of the constitutive ethylene-response phenotype of *etr1-9;ers1-3 *by transformation with wild-type *ETR1 *(*tETR1*). Phenotypes of 4-day-old seedlings are shown for a wild-type control, the *etr1-9;ers1-3 *mutant line, and two independent lines of *etr1-9;ers1-3 *transformed with *tETR1*.

ERS1 and ETR1 are closely related at the sequence level and, together, make up subfamily 1 of the receptors [3,6,4]. To gain information on how subfamily 1 receptors contribute to ethylene signaling, we constructed double mutant *etr1;ers1 *combinations by crossing *ers1-3 *with *etr1-7 *as well as with *etr1-9*. Effects of the mutations upon the triple-response phenotype of dark-grown seedlings were examined. As shown in Fig. 3A, the triple response of wild-type Arabidopsis seedlings to ethylene is characterized by inhibition of root and hypocotyl elongation, an exaggerated apical hook, and a thickening of the hypocotyls [23,24]. These features contrast sharply with the etiolated phenotype observed in dark-grown seedlings exposed to air. The single *etr1 *and *ers1 *mutant seedlings displayed phenotypes similar to wild type when grown in the absence of ethylene (in air) (Fig. 3A). However, double mutants of *etr1 *with *ers1 *displayed a constitutive ethylene-response phenotype when grown in the absence of ethylene. Significantly, the *etr1-7;ers1-3 *and the *etr1-9;ers1-3 *double mutants displayed a more pronounced phenotype (shorter hypocotyl and exaggerated apical hook) than the *etr1-7;ers1-2 *double mutant. This is consistent with our hypothesis that *ers1-2 *is a partial LOF allele and that *ers1-3 *is a true null allele. Both the *etr1-7;ers1-3 *and *etr1-9;ers1-3 *mutants displayed a similar constitutive ethylene-response-like phenotype, consistent with the newly identified *etr1-9 *being a null allele like the previously characterized *etr1-7*.

We confirmed that the severe constitutive ethylene-response phenotype of *etr1-9;ers1-3 *was due to lack of the receptors by transformation of this line with wild-type *ETR1 *(Fig. 3B). For this purpose, a genomic clone containing both promoter and coding regions of *ETR1 *was used. Transgenic lines were indistinguishable from a wild-type control when examined for growth in the absence of ethylene (in air).

### Characteristics of *etr1;ers1-3 *double mutant plants grown in the light

**Figure 4:**
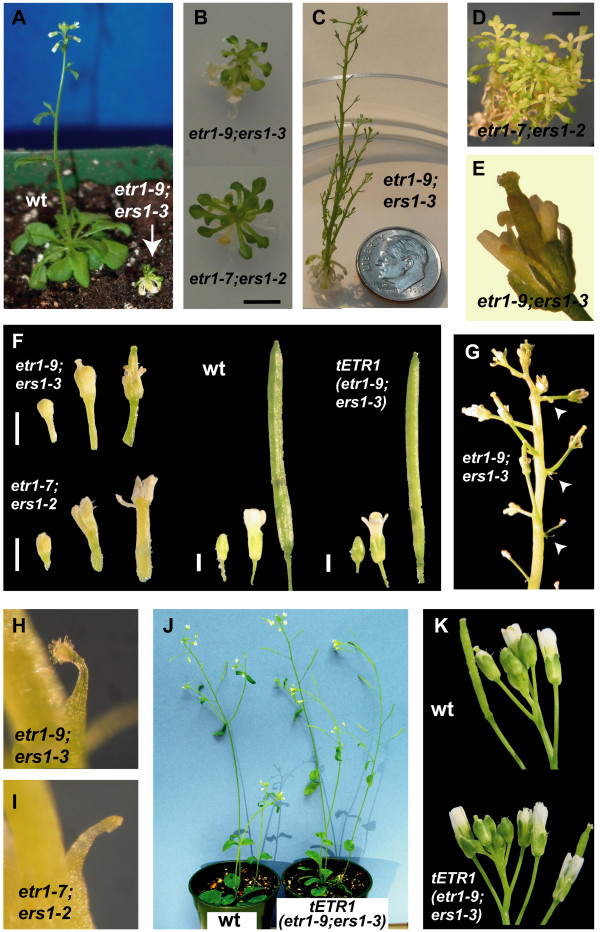
Light-grown *etr1-9;ers1-3 *mutant plants display multiple effects upon growth and development. (A) Comparison of 5-week-old wild type (wt) and *etr1-9;ers1-3 *double mutant. (B) Comparison of 5-week-old *etr1-9;ers1-3 *and *etr1-7;ers1-2 *double mutants. Scale bar = 5 mm. (C) Inflorescence of 7-week-old *etr1-9;ers1-3 *mutant. Coin for scale = 18 mm. (D) 7-week-old *etr1-7;ers1-2 *mutant plant that has not bolted. Scale bar = 5 mm. (E) Flower of *etr1-9;ers1-3 *mutant plant, showing reduced sepals, petals, and stamens compared to the central carpels. (F) Floral phenotypes of adult plants. Flowers of equivalent age are shown. Note that the *etr1-7;ers1-2 *flowers arrest at an earlier developmental stage than the *etr1-9;ers1-3 *flowers. No defects in flower development are observed in *etr1-9;ers1-3 *transformed with wild-type ETR1 (tETR1). Scale bar = 1 mm. (G) Location of filamentous structures on the inflorescence of the *etr1-9;ers1-3 *mutant. (H) and (I) Close-ups of filamentous structures found on *etr1-9;ers1-3 *and *etr1-7;ers1-2 *mutants, respectively. (J) Phenotype of *etr1-9;ers1-3 *transformed with ETR1 (tETR1) in comparison to wild type (wt). Six-week-old plants are shown. (K) Inflorescence of the *etr1-9;ers1-3 *mutant transformed with ETR1 (tETR1) compared to wild type. Note that the transformed mutant no longer produces filamentous structures.

From this point forward in the text, when we refer to *etr1;ers1-3 *mutants we are referring to both the *etr1-7;ers1-3 *and *etr1-9;ers1-3 *mutants, with the intent here to indicate that both exhibited the same phenotype. We refer to the specific genotypes as appropriate to the experiment and where pictured in the figures. When grown in the light (Fig. 4A, B, C), the *etr1;ers1-3 *mutant plants were extremely reduced in stature as is found with a constitutive ethylene-response mutant, but with a severity greater than that found with the *ctr1 *mutant or any previously characterized receptor mutant combination including the double mutant *etr1-6;ers1-2 *and the quadruple mutant *etr1;etr2;ein4;ers2 *[13]. Leaves of the *etr1;ers1-3 *mutants were epinastic (Fig. 4A, B, C). Epinasty is a well-documented effect of ethylene and has been previously observed in *ctr1 *mutants and higher order ethylene-receptor mutants [13,25].

**Figure 5:**
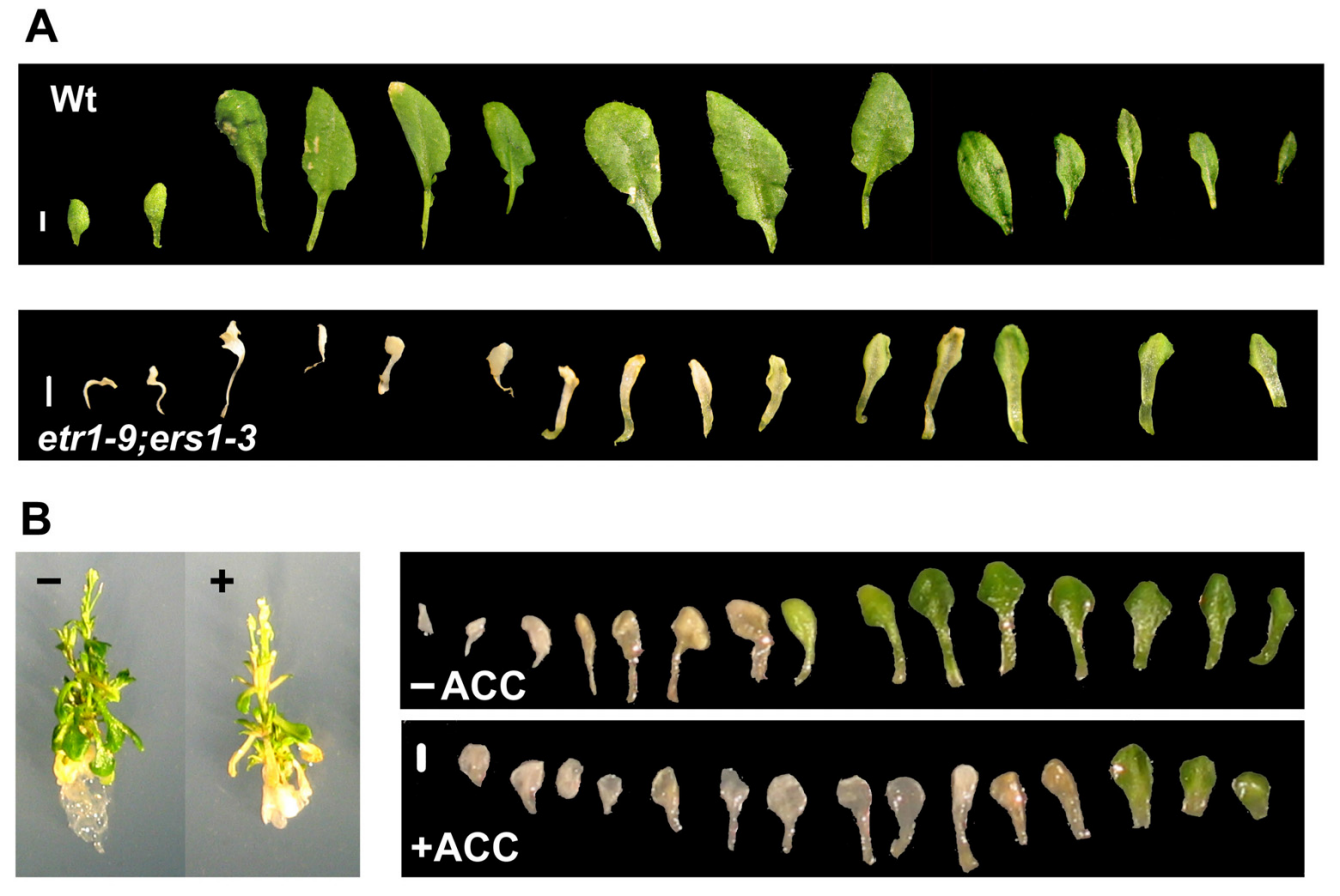
Accelerated senescence of leaves from *etr1;ers1 *mutant plants. A, Leaves of five-week-old *etr1-9;ers1-3 *and wild-type plants showing differences in senescence. B, Increased senescence of *etr1-7;ers1-2 *leaves upon treatment with the ethylene precursor ACC. Five-week-old plants are shown, the ACC treatment (+) being for 27 days with 50 μM ACC. Scale bars equal 1 mm.

Leaves of the *etr1;ers1-3 *double mutants senesced prematurely when compared to wild type (Fig. 4A, B, C and Fig. 5). An effect upon leaf senescence has not been previously reported with other receptor mutant combinations but is consistent with the role of ethylene as a regulator of the senescence response in plants. Analysis of the weaker *etr1-7;ers1-2 *mutant also revealed a degree of premature senescence but not as pronounced as that observed with the *etr1;ers1-3 *mutants (Fig. 5). By treatment of the weaker *etr1-7;ers1-2 *double mutant with the ethylene precursor ACC, we were able to induce a level of leaf senescence comparable to that observed with *etr1-9;ers1-3 *(Fig. 5). In addition, the ACC treatment resulted in an inhibition of root growth and decreased production of leaves from the axillary meristems of *etr1-7;ers1-2*, so that the overall morphology of the plant appeared more similar to that of *etr1-9;ers1-3*. Our ability to phenocopy features of *etr1-9;ers1-3 *by ACC treatment of *etr1-7;ers1-2 *is consistent with the *etr1-9;ers1-3 *double mutant showing a more pronounced ethylene response phenotype than that observed in wild type as well as *etr1-7;ers1-2 *plants.

The ability of the *etr1;ers1-3 *mutant plants to bolt was affected by the light conditions used for growth. The *etr1;ers1-3 *mutant plants died without bolting when grown at 120 μE light, producing greater than wild-type numbers of leaves under this growth condition. Previously, the *etr1;etr2;ein4;ers2 *quadruple LOF mutant was often observed to wilt and die before bolting, potentially for similar reasons [13]. Improved growth of *etr1;ers1-3 *occurred when the plants were grown under constant light at light levels below 60 μE. Under these growth conditions, the timing for the transition from vegetative to reproductive growth for the *etr1;ers1-3 *double mutants occurred similarly to wild type (*etr1-7;ers1-3 *produced 15 rosette leaves compared to 13.3 for wild type). The wild-type-like bolting time of the *etr1;ers1-3 *mutants differs from that found with the *etr1-7;ers1-2 *mutant, which is delayed in bolting and produces many additional leaves from axillary meristems (Fig. 4D) [19]. But, as described in the previous paragraph, by optimizing growth conditions and treating *etr1-7;ers1-2 *with the ethylene precursor ACC (Fig. 5B), we could partially phenocopy *etr1-9;ers1-3 *indicating that this difference in bolting may arise due to differences in the level of signaling through the ethylene pathway.

Although the *etr1;ers1-3 *mutants flowered, they had severely reduced fertility and altered floral morphology with an apparent decrease in the size of some floral organs compared to wild type (Fig. 4E, F). The carpels appeared normal in size, but the outer whorls of stamens, petals, and sepals were reduced in size. Floral buds appeared as though they had progressed through stage 13 as defined according to [26]. The flowers had open buds and visible petals, but did not progress to later stages during which petals and stamens elongate above the stigamatic papillae. Most flowers contained 4 stamens instead of the usual 6. In most cases, no seeds were produced. In a few cases, however, small siliques were made containing seeds of reduced size, indicating that the double mutants could self-pollinate. We obtained 20 seeds from *etr1-7;ers1-3 *and 6 seeds from *etr1-9;ers1-3 *plants, but none of the seeds germinated. These effects upon flower development are similar to those reported for an *etr1-6;ers1-2 *double mutant [19], but not quite as severe as those found in the *etr1-7;ers1-2 *double mutant which often did not progress beyond stage 11 (Fig. 4F) [19].

We also observed the formation of novel organs at the base of the pedicel of *etr1;ers1-3 *mutants (Fig. 4G, H). These appeared at the position where a subtending leaf (bract) is formed in many non-Arabidopsis plant species. The organs appeared filamentous in morphology, suggesting that not only is a developmental pathway being activated that is normally suppressed in Arabidopsis, but that there may be homeotic conversion of the organ produced from a leaf-like to a flower-like structure. We also found similar filamentous organs when we examined *etr1-7;ers1-2 *under optimized growth conditions that promoted bolting (Fig. 4I). The formation of the same organs in *etr1-9;ers1-3 *and *etr1-7;ers1-2*, which were generated with independent insertion mutations, confirms that loss of the receptors is the basis for formation of the filamentous organs.

Expression of a wild-type *ETR1 *transgene in the *etr1-9;ers1-3 *mutant background rescued all the phenotypes we observed in the *etr1-9;ers1-3 *double mutant. The transgenic lines were indistinguishable from wild type in terms of stature, leaf senescence, and flower development. In addition, the transgenic plants no longer made the filamentous organs at the base of the pedicel. These data are thus consistent with all the phenotypes found in the double mutant as having originated from lack of the two receptors ETR1 and ERS1.

### Ethylene responsiveness of the *etr1-9;ers1-3 *double mutant

The constitutive ethylene-response phenotype for the *etr1;ers1-3 *double mutants is stronger than that observed for any previous receptor mutant combination. Because both ETR1 and ERS1 belong to subfamily 1, this raises the question as to whether the ethylene response phenotype has reached maximal levels in the double mutant. If so, then this would imply that the subfamily 1 receptors are absolutely required for mediation of the ethylene response. However, if the *etr1;ers1-3 *mutant still displays an ethylene response, then this would indicate that subfamily 2 receptors are able to contribute to the ethylene response independently of subfamily 1 receptors. We therefore examined the ethylene response in the *etr1-9;ers1-3 *double mutant background.

**Figure 6:**
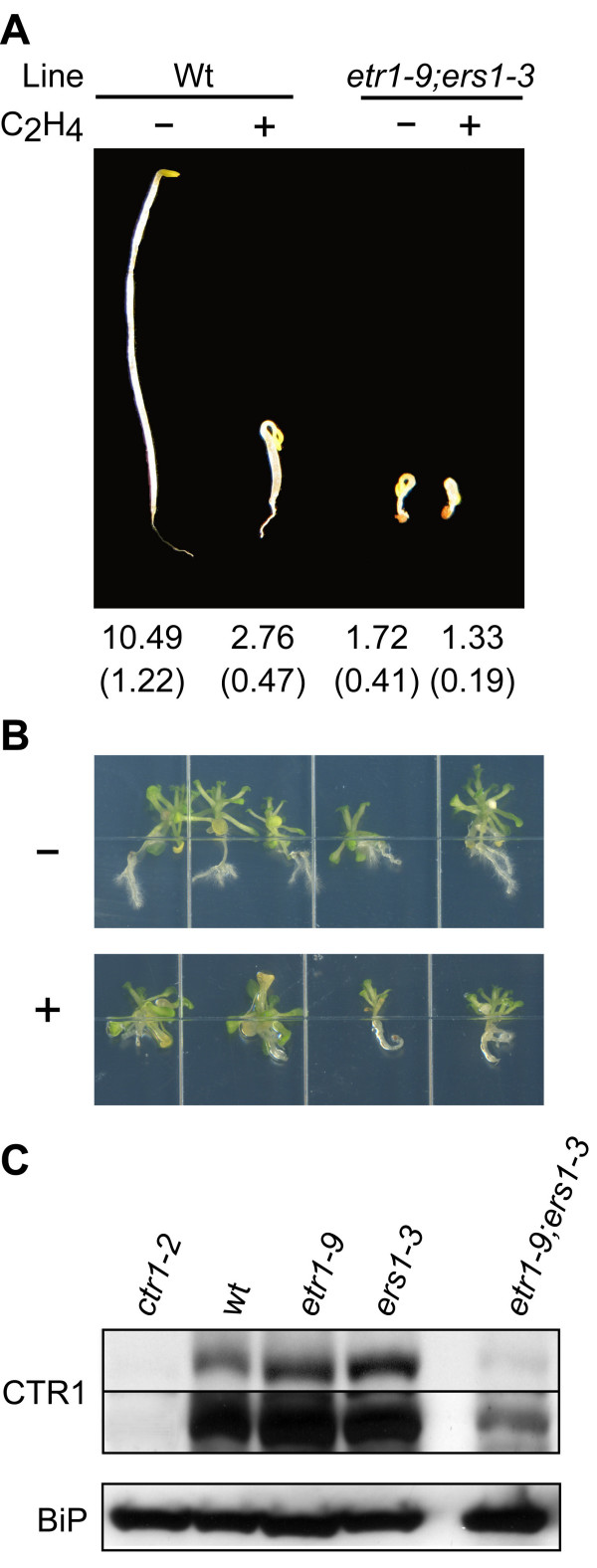
Ethylene response of the *etr1-9;ers1-3 *double mutant. (A) Effect of ethylene (10 μL/L) upon 4-day-old dark-grown seedlings (based on >20 seedlings per treatment). Mean hypocotyl lengths are given in millimeters with SD in parentheses. AVG (5 μM) was included in growth media to inhibit ethylene biosynthesis by the seedlings. (B) Effect of 50 μM ACC upon root and shoot growth of light-grown seedlings. Seedlings are 25-days old, the ACC treatment for 18 days. (C) Levels of CTR1 associated with microsomes based on immunoblot analysis. Two exposures of the CTR1 blot are shown. BiP is included as a loading control.

In dark grown seedlings, we observed a small but significant difference (P < 0.01 in Student's *t *test) between the hypocotyl lengths of *etr1-9;ers1-3 *seedlings grown in the presence or absence of 10 μL/L ethylene (Fig. 6A). However, due to the small effect found in the dark-grown seedlings, we also examined light-grown seedlings grown in the presence or absence of the ethylene precursor aminocyclopropane (ACC) (Fig. 6B). ACC treatment did not significantly affect rosette leaf size but did result in a reduction in root growth of the mutant seedlings. This result suggests that subfamily 2 receptors can act independently of subfamily 1 receptors in transmission of the ethylene signal for at least some growth responses.

Ethylene signaling by the receptors occurs through regulation of the kinase CTR1. Previous work has demonstrated that CTR1, which contains no transmembrane domains, is membrane localized due to its physical association with the ethylene receptors [15]. We could still detect a reduced level of CTR1 in the membranes of *etr1-9;ers1-3 *(Fig. 6C), consistent with subfamily 2 receptors being able to bind CTR1 independently of the subfamily 1 receptors. This result suggests that the residual level of ethylene response found in *etr1-9;ers1-3 *is mediated by the regulation of CTR1 activity through subfamily 2 receptors. In addition, as previously found when examining the levels of membrane-associated CTR1 in the *etr1-7 *single mutant [15], we found increased levels of membrane-associated CTR1 in the *etr1-9 *and in the *ers1-3 *single mutants. This result suggests that loss of a subfamily 1 receptor may be partially compensated for by the binding of additional CTR1 to the remaining ethylene receptors.

## Discussion

In *Arabidopsis*, ethylene signaling is mediated by a receptor family consisting of five members. LOF mutations have been isolated in the genes encoding all five receptors and these mutations have been used to assess the contribution of each member to the plant ethylene response [13,17-19,22]. Our results indicate that the previously isolated T-DNA insertion mutation *ers1-2 *is a partial LOF allele not a null allele of *ERS1*. Northern-blot analysis indicated that although there was a substantial reduction in the message level, full-length *ERS1 *transcripts were still detectable in the *ers1-2 *background [17]. The T-DNA insertion in the 5'-UTR of *ERS1 *in the *ers1-2 *allele could potentially result in a null allele because the altered *ERS1 *transcript contains additional upstream start codons arising from the T-DNA sequence [18]. However, the wild-type *ERS1 *gene contains two upstream start codons in the 5'-UTR, which apparently do not disrupt the correct transcription of the gene. Consistent with *ers1-2 *being partial LOF rather than a null allele, we found that the *etr1-7;ers1-3 *and *etr1-9;ers1-3 *double mutants displayed a stronger constitutive ethylene-response phenotype than the *etr1-7;ers1-2 *double mutant. The isolation of the *ers1-3 *allele allows for a reassessment of the contribution of *ERS1 *and the subfamily 1 receptors to ethylene signaling.

Previous work has shown that ethylene receptors are negative regulators and function redundantly in ethylene signaling [13]. Our results are consistent with this model, and indicate that the subfamily 1 receptors play a greater role in signaling than the subfamily 2 receptors. First, single LOF mutations in either subfamily 1 receptor *ETR1 *or *ERS1 *resulted in a slight increase in ethylene sensitivity, whereas single LOF mutations in subfamily 2 receptors were indistinguishable from wild type [13]. Second, the *etr1;ers1-3 *double mutants displayed a strong constitutive ethylene-response phenotype, greater than that observed with any previously characterized mutant combination including a quadruple LOF mutant of ETR1 and the subfamily 2 receptors [13]. In contrast, a triple mutant of subfamily 2 receptors is primarily distinguished by an increase in its ethylene sensitivity such that it exhibits a partial triple response phenotype due to its responsiveness to endogenous ethylene levels in the seedling [22]. Third, the pronounced effect of the *etr1;ers1-3 *double mutants on signaling was not dependent upon growth in the light, as found in a previous study of the *etr1;ers1-2 *mutant [19], indicating that a predominant role for subfamily 1 receptors may be a general mechanistic feature of ethylene signal transduction.

Our results also suggest that signaling by the subfamily 2 receptors may be partially dependent upon the subfamily 1 receptors. It was previously found that when LOF mutations of subfamily 2 members are combined with the LOF *etr1-7 *mutation, the higher order loss-of-function mutants display a progressively stronger constitutive ethylene response phenotype [13]. Thus under these conditions of analysis, where subfamily 1 member *ERS1 *is still present, the subfamily 2 members appear to make significant contributions to signaling. We find, however, that the *etr1-9;ers1-3 *mutant shows a strong constitutive ethylene response phenotype which is only minimally affected by ethylene treatment, suggesting that there is little additional signaling by the subfamily 2 receptors under conditions when both subfamily 1 receptors are missing. Consistent with the hypothesis that the function of subfamily 2 receptors may be partially dependent on subfamily 1 members is the finding that the dominant ethylene-insensitive mutant of ETR2 (*etr2-1*) is less effective in a *etr1-7 *background that lacks ETR1 than in a wild-type background [22], as well as the finding that the *etr1-7;ers1-2 *mutant cannot be complemented by a subfamily 2 receptor [18].

Examination of the *etr1;ers1-3 *double mutants reveals physiological effects upon plant growth and development not previously noted in the examination of higher order mutant combinations of the ethylene receptors, in particular the extremely reduced stature of the seedlings, the premature leaf senescence, and the development of the filamentous structures at the base of the flower. In addition, we are able to confirm some of the developmental defects found in the *etr1;ers1-2 *mutants, in particular the floral organ defects such as reduced organ size and numbers, as well as the reduced fertility. This is important because *ers1-2 *was generated from a T-DNA that contains part of the AP3 promoter, which has been found to result in fertility problems in some of the insertion lines [20]. Expression from the AP3 promoter may account for some of the variability in the point at which flower development terminated in the previously characterized *etr1;ers1-2 *lines, in particular the termination of flower development in stage 11 of the *etr1-7;ers1-2 *mutant [19], which is earlier than the stage 13 termination observed in the *etr1-6;ers1-2 *mutant [19] as well as the newly isolated *etr1;ers1-3 *mutants.

Light quantity and quality affected growth of the *etr1;ers1-3 *double mutants. The *etr1;ers1-3 *double mutants did not flower under standard light conditions for Arabidopsis growth, potentially due to stress responses, which resulted in bleaching of the leaves and a cessation of growth prior to bolting. But, when the light intensity was reduced, the transition from vegetative to reproductive growth of *etr1;ers1-3 *occurred similarly to wild type. Interestingly, the previously characterized *etr1-7;ers1-2 *mutant shows a substantial delay in flowering time compared to wild type, as measured either by days to flowering (63 days compared to 22) or by the number of rosette leaves produced before flowering (25–45 more rosette leaves than wild type) [19]. Most of these additional leaves arise from axillary meristems, and under optimized growth conditions, *etr1-7;ers1-2 *did not exhibit as pronounced a delay in flowering, often producing similar numbers of leaves as wild type from the shoot apical meristem (Figure 5). The *etr1-7;ers1-2 *mutant still initiated more leaf formation than *etr1;ers1-3 *from the axillary meristems, but axillary leaf production was reduced by treatment with the ethylene precursor ACC, indicating this phenotype may arise from differences in activation of the ethylene signaling pathway in the different backgrounds. Overall, our results suggest that ethylene signaling interacts with light-dependent signaling pathways in several ways, both affecting the light stress response and the cues that regulate the transition from vegetative to reproductive growth.

The greater role played by the subfamily 1 receptors compared to the subfamily 2 receptors in ethylene signaling raises the question as to which features of subfamily 1 members are important for this role. Many features are shared between the two subfamilies. Both subfamily-1 and subfamily-2 ethylene receptors appear to have a similar affinity for ethylene [7] and localize to the ER membrane [15,27]. Thus, the most distinctive characteristic of the subfamily 1 receptors is that each possesses a functional histidine kinase domain [11,12]. To date only minor roles in signaling have been attributed to the histidine kinase activity [28,29] but, due to the lack of an *ERS1 *null allele, it has not been previously possible to eliminate histidine kinase activity of the receptors in the backgrounds examined. Thus, a reevaluation of the role of kinase activity in signaling is needed. Our results do indicate, however, that histidine kinase activity is not an absolute requirement for an ethylene response, because we still detected a weak ethylene response in the *etr1-9;ers1-3 *double mutant, primarily in the effect of ethylene upon root growth. Plants thus contain an ethylene signaling pathway that does not require the kinase activity of the subfamily 1 receptors, although the degree to which this pathway contributes to signaling is not known at this point.

The interaction between the ethylene receptors and the Raf-like kinase CTR1 represents an alternative mechanism by which subfamily 1 receptors may play a greater role than subfamily 2 receptors in mediating the ethylene response. *CTR1 *is a negative regulator of ethylene signaling and loss-of-function mutations in *CTR1 *result in a constitutive ethylene response phenotype [16,25]. CTR1 physically interacts with the receptors and, as a result, localizes to membranes of the endoplasmic reticulum [15]. Our results demonstrate that loss of subfamily 1 receptors results in a decrease in the levels of CTR1 at the membrane, which correlates with the strong constitutive ethylene response phenotype observed in the *etr1;ers1-3 *double mutants. We still observe some CTR1 present on the membrane, however, indicating that subfamily 2 is sufficient for localization of a proportion of CTR1 to the membrane. These data, coupled with our previous analysis of receptor LOF mutants [15], indicate that both subfamily 1 and subfamily 2 are capable of independently localizing CTR1 to the membrane. Thus the difference in roles of the two receptor subfamilies is not simply due to one subfamily interacting with CTR1 and the other not.

Two observations, however, suggest that the interaction of CTR1 with subfamily 1 receptors is not necessarily equivalent to its interaction with subfamily 2 receptors. First, when analyzed by two-hybrid analysis, subfamily 1 members ETR1 and ERS1 both exhibit a stronger interaction with CTR1 than does subfamily 2 member ETR2 [14,22]. Second, the level of membrane-associated CTR1 does not always correlate with the level of signaling through the ethylene pathway. Significantly, single null alleles in either of the subfamily 1 members *ETR1 *and *ERS1 *actually result in a higher level of CTR1 associated with the membrane (Figure 6). This effect is only found with LOF mutations in subfamily 1, not with subfamily 2 [15], and may serve to partially compensate for the loss of the subfamily 1 receptor. But, even with the higher levels of CTR1, the *etr1 *and *ers1 *mutant seedlings still exhibit increased sensitivity to ethylene (Figure 2) rather than the decreased sensitivity predicted if the ethylene response were directly proportional to the amount of CTR1 present. Thus, the predominant contribution of subfamily 1 receptors to ethylene signaling may arise from their possessing a greater effectiveness at maintaining CTR1 in an active state compared with the subfamily 2 receptors.

## Conclusion

**Figure 7:**
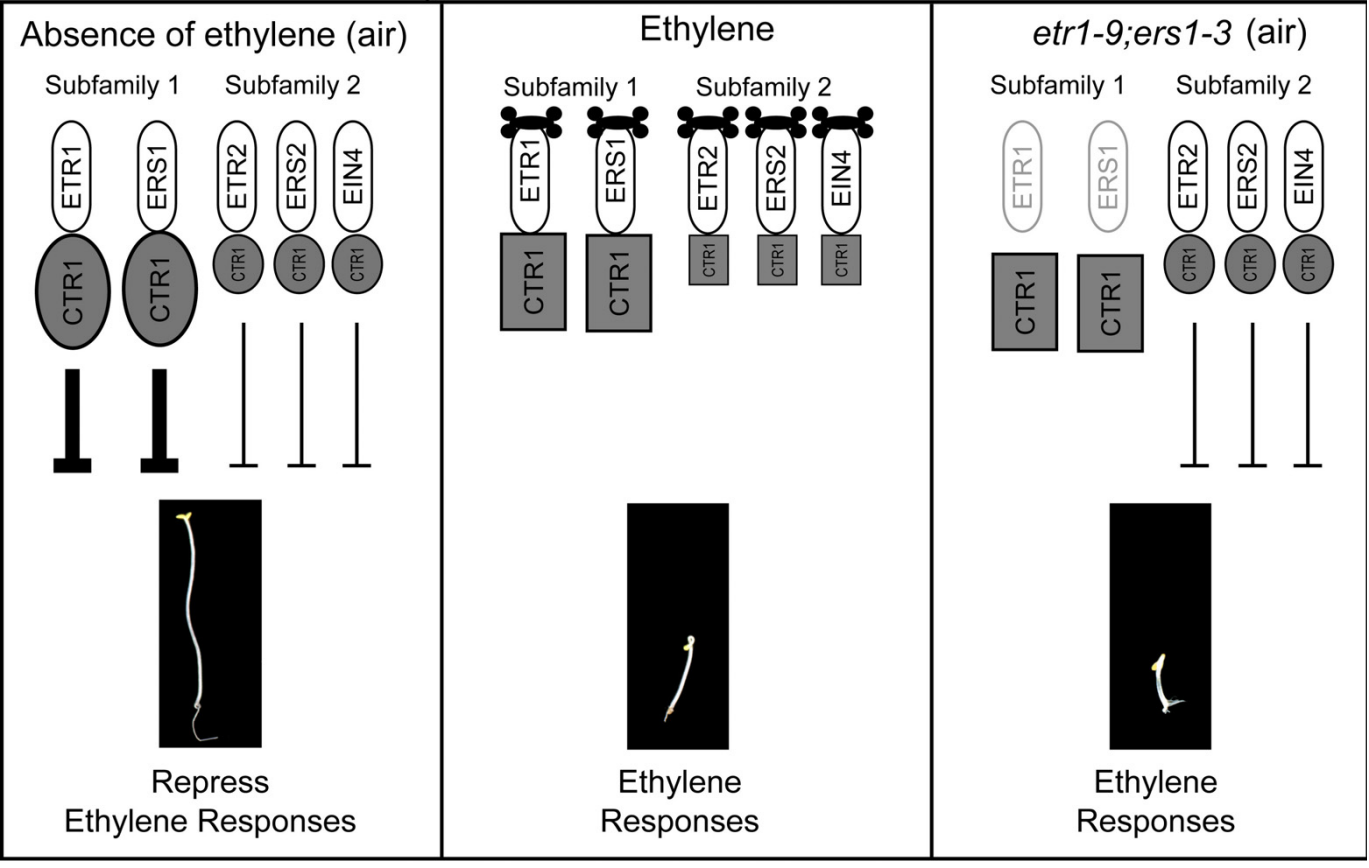
Model for signaling by ethylene receptors. CTR1 (shown in gray) interacts with all five receptors, but subfamily 1 receptors activate CTR1 to a greater extent than subfamily 2 receptors. In the absence of ethylene (in air), the kinase domain of CTR1 actively represses ethylene responses. Ethylene binding to the receptors induces a conformational change in CTR1 that reduces its kinase activity, thereby relieving repression of the ethylene response pathway. Loss of subfamily 1 receptors (the *etr1;ers1 *mutant), leads to the stimulation of ethylene responses because there is not enough active CTR1 to suppress the pathway. In the figure, active CTR1 is indicated by a circle and inactive CTR1 by a square; the size of the CTR1 symbol indicates its relative contribution to signaling.

A model for ethylene signaling that incorporates the results from the analysis of the *etr1;ers1-3 *mutant is shown in Figure 7. Our data are consistent with a greater role for ETR1 and ERS1 (subfamily 1) in the regulation of ethylene signaling as compared to the other three members of the ethylene receptor family (subfamily 2). Loss of either subfamily 1 receptor is partially compensated for by the other, but loss of both together results in a strong constitutive ethylene-response phenotype. Previous analysis suggested that the predominant role for subfamily 1 might be limited to light-grown plants [19], but our analysis of the *etr1;ers1-3 *mutants indicates that the model also applies to dark-grown seedlings, suggesting that this model may apply to most aspects of ethylene signal transduction. The *etr1;ers1-3 *double mutant is only minimally affected by ethylene, but the residual response detected indicates that subfamily 2 receptors are capable of initiating a response independently of subfamily 1. As indicated in the model, the greater role for subfamily 1 receptors compared to subfamily 2 receptors may lie in an enhanced ability to stimulate the kinase activity of CTR1.

## Methods

### Plant growth conditions

To examine the triple response of seedlings to ethylene [30,31], seeds were grown on Petri plates. The inhibitor aminoethylvinyl-glycine (AVG) was included in growth media at a concentration of 5 μM as indicated to inhibit ethylene biosynthesis by seedlings. Plates were placed in sealed containers with 0 to 1000 μL/L ethylene. To examine seedlings growing in the absence of ethylene, hydrocarbon-free air was passed to remove any ethylene synthesized by the seedlings. All containers were kept in the dark at 22°C. Seedlings were examined after 3.5 or 4 days of growth, time 0 corresponding to the time when the plates were removed from 4°C and brought to 22°C. To measure hypocotyl length, seedlings were grown on vertically oriented square plates so as to simplify later scanning of the plates. Seedlings were scanned and hypocotyl length measured in NIH Image (version 1.6, National Institute of Health, Bethesda, MD).

To examine growth in the light, the *etr1;ers1 *mutants were grown at 22°C under constant light in Magenta cubes (Magenta Corporation, Chicago, IL) containing Murashige and Skoog basal media with Gamborg's vitamins (pH 5.75; Sigma, St. Louis), 1% (w/v) sucrose, and 8% (w/v) agar. Optimal growth was observed under reduced light intensity (below 60 μE), when using standard fluorescent bulbs augmented with 18,000 K fluorescent bulbs (e.g. Aqua-Glo, Rolf C. Hagen Corp., Mansfield, MA). For treatment with ACC, segregating seedlings were initially grown under standard conditions so as to identify the double mutant by phenotype and the seedlings then transferred to media containing 50 μM ACC for the times indicated.

### Identification and genetic analysis of T-DNA insertion mutant alleles in *ERS1 *and *ETR1*

Both *ers1-3 *and *etr1-9 *were isolated from Wisconsin Basta population at the Arabidopsis Knockout Facility at the University of Wisconsin-Madison [20], which contains 72,960 BASTA-resistant lines in the ecotype Wassilewskija (WS) transformed with an activation-Tag vector, pSK1015 [32]. The *ers1 *mutant was identified with a PCR primer for the T-DNA left border (5'-CATTTTATAATAACGCTGCGGACATCTAC-3') and an *ERS1*-specific primer (5'-CAGAGAGTTCTGTCACTCCTGGAAATGGT-3'). Plants containing the wild-type *ERS1 *gene were identified by use of PCR with the above *ERS1 *primer and a second *ERS1*-specific primer (5'-CACAACCGCGCAAGAGACTTTAGCA ATAGT-3'). The *etr1-9 *mutant was identified by a PCR-based method using an *ETR1*-specific primer (5'-GCGGTTGTTAAGAAATTACCCATCACACT-3') and the same T-DNA left border primer as described above. Plants containing the wild-type *ETR1 *gene were identified by use of PCR with the above *ETR1 *primer and a second *ETR1*-specific primer (5'-ATCCAAATGTTACCCTCCATCAGATTCAC-3'). PCR conditions for identification of the T-DNA inserts included: preheating at 96°C for 8 min (hot-start); 94°C for 15 sec, 60°C for 30 sec, 72°C for 2 min (40 cycles); with a final extension at 72°C for 4 min.

To generate the *ers1;etr1 *double mutants, the *ers1-3 *mutant was crossed to the *etr1-7 *and *etr1-9 *mutants. For genotyping the *ers1-2*,*ers1-3*, and *etr1-9 *mutations, PCR was performed using the T-DNA left-border primer and the gene-specific primer as described above. The *etr1-7 *mutation was identified by PCR-based genotyping of F_2 _progeny from the crossed plants as described [13].

The *etr1-9;ers1-3 *double mutant was transformed with a previously described full-length *ETR1 *construct driven by its native genomic promoter [29]. The construct was introduced into *Agrobacterium tumefacians *strain GV3101 and used to transform an *etr1-9;ers1-3/+ *line by the floral-dip method [33]. Lines homozygous for both *etr1-9 *and *ers1-3 *were identified by PCR-based genotyping, and homozygous for the transgene based on the segregation of hygromycin resistance and by PCR-based genotyping.

### RNA isolation, Northern Blot, and RT-PCR analysis

Total RNA was isolated from 4-d-old *Arabidopsis *etiolated seedlings by a modified method of Carpenter and Simon [34] using TRIzol^® ^Reagent (Gibco BRL, Grand Island, NY). RNA was separated on 1% (W/V) agarose gels using the NorthernMax-Gly system (Ambion, Austin, TX). Separated RNAs were transferred to the MagnaCharge nylon membrane (GE Osmonics, Minnetonka, MN) by the capillary method and fixed by UV-cross linking using GS Gene Linker UV Chamber (Bio-Rad Laboratories). Single-stranded DNA antisense probes were made using primers designed to anneal at the 3' end of an ERS1 cDNA clone (5'-ACTAGTGACTGTCACTGAGAA-3') or an ETR1 cDNA clone (5'-ATCCAAATGTTACCCTCCATCAGATTCAC-3'). Radio-labeled probes were made and stripped between hybridizations by using the Strip-EZ PCR system from Ambion according to the manufacturer's instructions. Radioactivity was imaged and quantified by phosphor imaging with a Molecular Imager FX (Bio-Rad Laboratories), using accompanying Quantity One software.

For RT-PCR analysis of the *etr1-9 *mutant, total RNA was extracted from plants, treated with RNase-free DNAse (Invitrogen), and used to make first strand cDNA using Superscript III First Strand Synthesis System for RT-PCR (Invitrogen) according to the manufacturer's instructions. Primers were designed to span introns so as to distinguish cDNA amplification products from genomic DNA amplification products. The primers used were ETR1-5'F (5' CGTGGAGTATACGGTTC 3') and ETR1-5'R (5' CTGGTGCAAGATTTAGTGTGATG 3') for amplification of a product 5' to the site of the T-DNA insertion, and ETR1-3'F (5' CATACCGAAAGTTCCAGCCATTC 3') and ETR1-3'R (5' CAAGCATCCATAACTCGATCCAAATTC 3') for amplification of a product 3' to the site of the T-DNA insertion. After 25 cycles, the PCR products were examined by gel electrophoresis and EtBr staining. Primers specific for ubiquitin, UBQ-SENSE (5'-GTGGTGCTAAGAAGAGGAAGA-3') and UBQ-ANTI, (5'-TCAAGCTTCAACTCCTTCTTT-3'), were used as a control.

### Immunoblot analysis

To isolate membrane proteins, plant tissue was homogenized in extraction buffer (50 mM Tris, pH 8.5, 150 mM NaCl, 10 mM EDTA, 20% [v/v] glycerol). As protease inhibitors, 1 mM phenylmethylsulfonyl fluoride (PMSF), 1 μg/mL pepstatin, 10 μg/mL leupeptin, and 10 μg/mL aprotinin, were included to prevent protein degradation. The homogenate was filtered through Miracloth (Calbiochem-Novobiochem, San Diego, CA), and then centrifuged at 8,000 g for 15 minutes. The supernatant was centrifuged at 100,000 g for 30 min, and the membrane pellet then resuspended in resuspension buffer (10 mM Tris, pH 7.5, 150 mM NaCl, 1 mM EDTA, and 10% [v/v] glycerol) with protease inhibitors. Procedures described above were all done at 4°C. Protein concentration was then determined by a modification of the Lowry assay [35] in which samples were treated with 0.4% (w/v) sodium deoxycholate [36]. Bovine serum albumin was used in preparing a standard curve. For the experiment in Figure 1, 4-d-old dark-grown seedlings were used as the membrane source. For the experiment in Figure 6, seedlings grown in liquid culture were used as the membrane source [15]; the double mutant seedlings were initially selected on Petri dishes based on phenotype then transferred to liquid culture.

Membrane proteins were mixed with 2× SDS-PAGE loading buffer [125 mM Tris-HCl (pH 6.8), 4% (w/v) SDS, 20% (v/v) glycerol, 0.01% bromphenol blue] containing 100 mM dithiothreitol as a reducing agent. After incubation at 50°C for 1 hour, proteins were separated by SDS-PAGE using an 8% (w/v) polyacrylamide gel  [37]. Precision Plus Protein *all blue *Standards were included as molecular weight markers (Bio-Rad Laboratories). Separated proteins were electro-transferred to Immobilon nylon membrane (Millipore, Bedford, MA). Immunoblot analyses were conducted by use of the anti-ETR1(165–401), anti-ETR1(401–738), and the anti-CTR1 antibodies, with the anti-(H^+^-ATPase) and anti-BiP antibodies used as a membrane and soluble fraction loading controls, respectively [15,29]. Immunodecorated proteins were visualized by enhanced chemiluminescence detection according to the manufacturer (Pierce Chemical, Rockford, IL).

## Authors' contributions

XQ isolated and characterized mutations, transformed *etr1-9;ers1-3 *with the *ETR1 *construct, and helped to draft the manuscript. BPH identified homozygous *etr1-9;ers1-3 *lines containing the *ETR1 *construct, performed molecular and physiological characterizations of mutants and transformed lines, and helped to draft the manuscript. ZG analyzed CTR1 levels in the mutants. GES conceived and coordinated the study, performed physiological characterizations, and drafted the manuscript. All authors read and approved the final manuscript.
